# What information do patients want about their medicines? An exploration of the perspectives of general medicine inpatients

**DOI:** 10.1186/s12913-020-05911-1

**Published:** 2020-12-08

**Authors:** Amy Hai Yan Chan, Trudi Aspden, Kim Brackley, Hannah Ashmore-Price, Michelle Honey

**Affiliations:** 1 School of Pharmacy, Faculty of Medical and Health Sciences, University of Auckland, Grafton, Auckland, New Zealand; 2 Department of Pharmacy, Auckland District Health Board, Level 6, Auckland City Hospital, 2 Park Road, Grafton, 1023 Auckland, New Zealand; 3 School of Nursing, Faculty of Medical and Health Sciences, University of Auckland, Grafton, Auckland, New Zealand

**Keywords:** Patient preferences, Medicine, Hospital, Health services, Pharmacy, Information needs, Literacy

## Abstract

**Background:**

Medicines are one of the most common healthcare interventions, yet evidence shows patients often do not receive the information they want about their medicines. This affects their adherence and healthcare engagement. There is limited research exploring what information patients want about their medicines, from whom and in what format. The aim of this study was to determine the medicines information needs of patients admitted to the general medical service of a large New Zealand (NZ) hospital, and identify the barriers and enablers to meeting these needs.

**Methods:**

A descriptive exploratory approach using semi-structured interviews was used to understand the needs and preferences of patients for information about their regular medicines and the barriers and facilitators to obtaining this information. Patients admitted to a general medical ward at a large NZ hospital, aged 18 years and over, prescribed one or more regular medicines, and self-managing their own medicines prior to hospitalisation were included. Semi-structured interviews were conducted with each participant (*n* = 30) and transcribed, then analysed using a general inductive thematic analysis approach.

**Results:**

Five overarching themes captured the medicines information needs of patients: (1) autonomy; (2) fostering relationships; (3) access; (4) communication; and (5) minimal information needs. Patients desired information to facilitate their decision-making and self-management of their health. Support people, written information, and having good relationships with health providers enabled this. Having access to information at the right time, communicated in a clear and consistent way with opportunities for follow-up, was important. A significant portion of participants were satisfied with receiving minimal information and had no expectations of needing more medicines information.

**Conclusions:**

Although patients’ medicines information needs varied between individuals, the importance of receiving information in an accessible, timely manner, and having good relationships with health providers, were common to most. Considering these needs is important to optimise information delivery in general medical patients.

**Supplementary Information:**

The online version contains supplementary material available at 10.1186/s12913-020-05911-1.

## Background

Medicines are one of the most common healthcare interventions and play a significant role in patients’ health management. Yet research consistently shows that patients are dissatisfied with the amount and quality of medicines information they receive [1, 2]. Research has demonstrated discrepancies between the type of medicines information patients wish to receive and the type they receive from health providers [3–5]. This gap remains despite evidence highlighting that knowledge about medicines, and satisfaction with that knowledge, are important predictors of medicines adherence [6–8].

The need to deliver adequate medicines information to individuals to facilitate informed decision-making is particularly important with the shift towards patient self-management of their own health conditions and medicines [9]. To assist health providers to achieve this goal, a range of health information is available, however, there is limited literature on what information patients want, how they want to receive it, and when. A scoping review of medicines information needs of patients found that patients often desire information about adverse drug reactions and drug interactions [10]. However, scant information is available on how and when this information should be delivered. Furthermore, the review focused on specific drug information topics, which may have limited the scope of the findings. Other studies have explored general health information needs rather than medicines information [11], or have focused on specific populations, such as in individuals living with cancer [12, 13], post-myocardial infarction [14], asthma [15, 16], or post-surgery [17]. One study reported on information desired by general medicine patients at discharge, but did not explore the barriers or enablers to providing this information [18]. This study gave insight into where the medicine information gaps might be, including the need for information to be individualised according to needs and preferences, and delivered in both written and verbal formats [18].

The study aim was to determine the medicines information needs of patients admitted to the general medical service of a large urban New Zealand (NZ) hospital, and identify the barriers and enablers to meeting these needs. Specifically, the study aimed to determine what information about medicines patients want; how they would like this information provided during their hospital stay and after discharge; when they want the information delivered, and the barriers and enablers to accessing this desired medicines information.

## Methods

A descriptive exploratory approach [19] using semi-structured interviews was used to understand the needs and preferences of patients for information about their regular medicines. Patients admitted to a general medical ward, aged 18 years and over, prescribed and self-managing at home one or more regular medications, and able to converse in English were eligible for inclusion. Eligible participants were identified and referred to the research team by the hospital pharmacist. Purposive sampling was used to ensure that a variety of ages, genders, and ethnicities representative of the hospital catchment area, were included. The researcher approached identified patients to explain the research, invite participation, obtain written consent and arrange an interview time.

Semi-structured interviews were conducted at the patient bedside. The interview schedule (Supplementary material S1) was developed from previous literature on medicines and health information needs to address the aims of the study, and comprised a series of open-ended and closed questions as prompts. Information was also gathered about patient’s use of the internet to access medicines information; the findings from this latter enquiry have been reported more comprehensively previously [20] so are only reported here in the context of information needs for individuals under the general medical service.

Interviews were audio-recorded and transcribed verbatim. Each participant was assigned a unique participant code to de-identify the interview data. The transcripts were checked by the researcher to ensure accuracy of the transcriptions, then analysed for themes, facilitated by NVivo 11. A general inductive thematic analysis approach [21] was used to extract, describe and explain the medicine information needs and experiences of participants. The interviewer in the study completed the initial coding, assigning text within each transcript to emergent themes. These themes were then discussed with the whole research team, who reviewed the preliminary codes, and created a coding framework to facilitate the analysis of all remaining transcripts. Any disagreements were resolved by consensus discussion. The identified key themes were then further divided into sub-themes and confirmed with the whole research team. All participants were offered a $20 grocery voucher as a thank you. Any clinical questions or concerns were passed on to the nurse or pharmacist as appropriate. Ethical approval for this study was received from the NZ Health and Disability Ethics Committee (NZHDEC 16/NTA/49). All participants provided written informed consent to participate; all references and quotations from participants in this study are de-identified, and names used are pseudonyms.

## Results

Of the 40 patients approached for an interview over a three-month period, 30 patients (age range 18–71 years) consented and were interviewed, and their data analysed. Of the 10 patients who were not interviewed, four declined to participate, three were discharged prior to the interview time, and three were not able to provide informed consent. Table 1 summarises participant characteristics. Of the 30 interviews, two were incomplete due to the patient being discharged or the patient feeling too unwell to continue. The interviews lasted between 10 and 45 min.

**Table 1:** Demographics of study participants

	Number (%) interviewed
**Medical Ward**	
General Medicine	25 (83%)
Cardiology	4 (13%)
Respiratory	1 (3%)
**Ethnicities**	
New Zealand European	16 (53%)
Pacific	5 (17%)
Māori	5 (17%)
Asian	3 (10%)
African	1 (3%)
**Total**	**30**

### Key themes

Five overarching themes illustrate the medicines information needs of patients: (1) autonomy; (2) fostering relationships; (3) access; (4) communication; and (5) minimal information needs (Table 2).

**Table 2:** Summary of themes and sub-themes from the interview data relating to medicine information needs

Themes and definition	Sub-Themes
1. Autonomy Patients desire information to facilitate informed decision-making, understanding of their own care and that promote active participation in their health.	1.1Support people Enable (and can prevent) autonomy. 1.2 Written information Enables patients to understand their medicines and this promotes autonomy.
2. Fostering relationships Patients’ want to establish relationships with their healthcare providers and rapport is an enabler to sharing medicines information.	
3.Access Patients felt they needed to access more information. This includes via healthcare providers, their community, cultural support and the internet.	3.1 Timeliness Information needs to be given at the right time.
4. Communication Providers need to have effective communication skills, including being able to clarify information if it is not understood, avoiding jargon, and to ensure consistent, clear messages.	4.1 Providers Healthcare providers have a key role to play to provide information in a consistent and clear way. 4.2 Clarification Information needs to be repeated or followed up. 4.3Distractions Patients can get distracted by pieces of information that they are given
5. Minimal information needs Patients are satisfied with receiving minimal information and have low expectations of the health system.	

### Autonomy

This theme encompasses the right to receive and therefore the need for health providers to supply information deemed important to an individual and which may enable active participation in their health journey.

“*I just like to know, because this is my body, this is my life … coz [sic] it’s the right of every patient to be fully informed”* (39, Māori).

Participants expressed a desire for autonomy and being able to make their own choices, thus needing information from all health providers to empower them to be involved in their own health journey.

Participants wanted a variety of different medicines information ranging from basic information such as doses, to an in-depth description of mechanisms of actions. Participants rationalised the need for information to enable “*Understanding what the rationale is behind prescribing something, rather than just, you know, feeling like you’re just chucking something else down*” (48, NZ European). Most patients also preferred to receive medicines information directly from the prescriber, with some referring to nurse specialists specifically. Pharmacists were the second most commonly quoted preferred provider of medicines information *“I just think pharmacists know more about the medications, like side effects and all that kind of thing than doctors”* (23, Asian). Other sources of information included family members, pharmaceutical companies, websites, research articles, nurses, and other patients. Several participants explicitly stated that information could come from *“everybody including professors”*and that they *“don’t mind who gives it, as long as it’s correct”* and *“fully explained”*, then it *“doesn’t really matter”*.

Participants wanted to understand their health condition, the purpose of the treatment and what benefits they might see or experience. Specifically, participants wanted to know what they were being treated for and the effects of the medication. In many cases it was felt that these basic information needs were not currently being met: “*I didn’t really know, all I was told was just to take it”* (56, Māori). Some wanted information to determine whether the medication was working sufficiently to warrant continuing treatment: “*So that’s why I didn’t take it, because I could see no change, you know”* (43, Pacific).

#### Support

Autonomy encompasses the desire for support people to be involved when receiving medicines information. This was expressed by those who were elderly, or those who had limited ability to self-care. Many older participants were concerned about not hearing information, or “missing” information, especially if a lot of information was given at once. One participant stated *“I think from the point of view of an elderly person, it’s great to have somebody with you. ‘Cos you miss things, if you don’t hear them”* (82, NZ European).

The importance of involving the wider whānau (family) in healthcare was highlighted by many participants, particularly in Māori, Pacific and Asian participants: *“I do like to have another person … just so that if there was something that I missed, if they’re listening, that they can pick up on, and they can, you know, help me with it later”* (57, Pacific)*.* This is further discussed under the Access theme.

Some wanted support people present to help manage emergency medical situations, such as hypoglycaemia: “*… if anything happens to me, and I’m out with them [support people] … if something happens, they know what to do”* (19, NZ European). Conversely, some felt that they would lose their independence if a support person also received the information: *“If there was a slight [problem], one of them might say, come and live with us … so no, no, I love my independence”* (85, NZ European).

#### Written information enabling autonomy

Written information enables patients to understand their medicines and therefore promotes autonomy. Many were concerned about forgetting, especially if a lot of information was given at once, or if they needed to communicate the information to someone else. Participants believed written information enabled efficient transfer of information and was a supportive resource to refer to: *“I’ve got a bad memory now, so, … I need back up”* (42, Māori).

Some felt that written information promoted deeper understanding as more detail can be given: *“And then he said, read it again, like when you get home, because you might understand it a bit more when reading it over a second time”* (19, NZ European).

*“There needs to be easy, or logical, and intuitive access to further information, layers of information”* (39, Māori).

### Fostering relationships

Patients’ desire to establish relationships with their healthcare providers highlighted the importance of rapport when sharing medicines information. Some discussed how familiarity with a provider was beneficial: *“I like it when the pharmacist says to me, “You do know that you can’t take that with so and so”, because they know your medication”* (48, NZ European), as it was easier to share information when they had established rapport: *“I think just feeling comfortable enough to ask”* (24, NZ European). These relationships were beneficial and many talked about staying with a particular provider to maintain continuity of care. *“I’ve been going to the same chemist for 50 years; I’ve made a point of it, even though the doctor has moved, I still go back”* (85, NZ European).

Patients talked about a lack of consistency being a barrier as it limited rapport and prevented the prescriber from understanding their individual needs, and meeting their information needs. *“I think sometimes if people don’t know you and don’t know your health literacy, they don’t want to give you lots”* (24, NZ European). Consistency was often mentioned as a problem in the hospital system as medicines information provision was given by different healthcare workers. “C*onsistency is a bit of a tricky thing. Yeah, because, especially if you’re in the hospital system, you may see your higher up doctor, and then you see all his underlings for the next 20 times, you know. And that is a little tricky, and it is a little hard, coz [sic] consistency is very lacking”* (55, NZ European).

### Access to medicines information

Some talked about the need for access to more information, including via a healthcare provider, community or cultural support or information from the internet [20]. Some participants expressed an interest in access to information, or someone to explain, complementary and alternative medicines, such as herbal medicines that were part of their traditional cultural practices, or simply a desire for” *natural options*”.

Participants with access to community, whānau (family) or cultural support felt that these resources helped them to get information about their medicines. One participant described how a local community support group for people with diabetes helped them to understand their condition better and to get information about how they can manage their condition:

*“Where we come from, we have whānau [family] meetings about coping with diabetes, and all that … just to be on track”* (37, Māori). This participant went on to say that holding the support group in a culturally appropriate community setting made the members more comfortable. *“Because most whānau [family] where we’re from, they don’t like visiting doctors. That’s why we did this marae [cultural meeting place] one. And then we bring the GPs and that to the marae, and it makes them feel more at home, sort of thing”* (37, Māori). Some highlighted that cultural concordance enables understanding of their medicines because *“They’re your own sort of people, they can understand where you’re coming from as well*” (53, Pacific). Interestingly, the influence of culture and family support was believed to be less important by some NZ European respondents: “*we’re not strong on culture of any description”* (85, NZ European).

Several used online support groups to gain information from someone with personal experience. Despite appreciating access to online resources, participants also enjoyed having unscheduled access to their usual provider to ask questions – either with a community pharmacist who was close by or their doctor and/or nurse via telephone, or email: *“Yeah, they normally respond back pretty quickly, which is always good. And with my doctor, I can always just call the main desk and with my doctor you just give them like your name, and then they normally put you through to your doctor*” (19, NZ European).

Participants described using the internet to access medicines information to meet needs that were unmet due to a lack of information received, or not understanding the information given. Participants described using the internet to access information written in a manner that they could understand, including in different languages. Participants used the internet as they found it easier to access than a healthcare professional. It was seen as particularly helpful while people are acutely unwell because it is *“much easier to access something from home”* (24, NZ European).

For many participants, although convenient the internet was not viewed as a trustworthy source of information. Participants discussed ‘Google doctoring’ but “*didn’t believe everything they read”*. Despite the validity of the information being doubted, some participants reported using the internet because it was their only source of medicines information. Some participants suggested that the usefulness and safety of using the internet as a source of medicines information could be improved by providing a trustworthy site affiliated or endorsed by a hospital or government department. *“If there was a guaranteed, maybe a site that was actually connected to the hospital would make me feel a little bit better, coz that’s what holds me back, I suppose”* (55, NZ European). The use of the Internet to access medicines information is discussed further elsewhere [20].

Limited consultation times with health providers prevented some participants from getting their medicines information needs met. Health providers were often described as” *busy*” and for many participants the limited consultation time was a barrier. This left patients feeling confused about their medicines, and reluctant to return to the provider: *“You see them for 10 minutes, it costs you [amount] and then you’re rushed in and rushed out and you’re going “Well, what?””* (28, NZ European).

#### Timeliness

This refers to the need for information to be given at the right or appropriate time for the patient. For most participants, this meant from the prescriber at the time of prescribing. Participants wanted the information prior to taking the medicine because it gave them time to process the information and understand it: “*Definitely before I start taking it. I want to have the opportunity to reason through why, why now, you know”* (39, Māori).

Some participants discussed the difficulties of information being provided when they were acutely unwell. In these circumstances participants still wanted to be given information, but acknowledged that follow up, or providing information in writing would be required to ensure retention and understanding.

*“That gets [to be] a big thing, because if you’re really sick you’re not going to take anything in. So maybe it should be written down for you, so you can read it afterwards. And then once you are more coherent, I suppose, it should be explained to you, but you should have information”* (55, NZ European).

#### Communication

Effective communication skills such as speaking slowly and more clearly, using” *everyday language*” and providing information in smaller chunks were considered necessary for sharing medicines information, particularly when responding to those with functional, cognitive or language challenges.

#### Provider

Participants described problems caused by the poor communication skills of their healthcare providers such as speaking too quietly or quickly for them: *“They all talk so like this (whispering). And I’m second guessing all the time”* (88, NZ European); too much terminology: *“Like medical terms and stuff”* (42, Māori); and too much information at once. “*Sometimes there’s too much information”* (42, Māori). Some who had experienced these difficulties had become confident at asking for what they needed. *“When I am stuck, I ask, you know, “Give me explanation other ways, you know …”* (41, African).

Participants raised the issue of how healthcare providers share information. Many assumed information about their conditions and treatments were shared between providers already *“coz [sic] they all work together*” (59, Pacific). Some were concerned about a lack of communication between their different healthcare teams, with concerns about interactions between medicines *“you’ve got a doctor, a cancer doctor over here, and you’ve got a heart one over here, he wouldn’t know what he’s prescribed, what I got, until later”* (56, Māori).

#### Clarification

Having opportunities to review and re-visit medicines information received was desirable for several reasons, such as reinforcing information, checking understanding and providing an opportunity to review the information and add to their knowledge by asking questions. Asking questions enabled understanding of medicines information, as one participant stated: *“When I started asking questions, it actually made more sense to me why he’s giving that to me”* (19, NZ European). Some participants wanted repetition of medicines information to refresh their memory and remind them about the purpose and benefits of their medication: *“But then, secondary, I would ask, you know, stuff of the pharmacist, just to refresh my memory”* (46, Asian).

For many participants written information provided a reminder of the information received, as *“It [information] hasn’t sunk in ‘til I go home, and it makes me think”* (51, Māori). For others, clarification was desired because they were not confident in their use of the medicine, mostly with non-oral medications, such as inhalers and injections. One participant prescribed intramuscular injections, stated “*I never know how give it, and sometimes it’s really painful. And I suspect I’m giving it wrong “*(63, NZ European). Patients felt embarrassed about not knowing the information, and were reluctant to ask for the information again: *“because by now they must think I’m just so stupid, she must have figured it out by now. But, no, I’m still struggling with my once a week”* (63, NZ European).

For some, knowing who to follow up with was important. *“I want to be clear, if I want to follow this up, which I will, and I’ve got questions, who do I go to?”* (39, Māori). Patients wanted more information and reassurance particularly when the medicine appearance, or dose, were changed. Some participants stated that as they were not notified about brand changes, they believed a dispensing error had occurred: “*Say it’s a big round pill, and all of a sudden you get this long sausage one, you know, you go, “Ohhh”, and you think you’ve got the wrong pills”* (56, Māori).

#### Distractions

Some patients described being distracted by trigger pieces of information. One patient described it as *“Sometimes you get stuck on one thing, you know, you think, “… I’ve got to take Prednisone, I’ve got to have a steroid injection”, and you don’t hear anything after that”* (80, NZ European). This idea was also described by patients who were given a lot of information at once. For these patients, written information and/or follow up was especially important so that *“At least you could take it home, have a quiet time, sit down, and read it, and then you’d understand”* (56, Māori).

### Minimal information needs

The theme of ‘minimal information needs’ encompasses the idea of patients being accepting of receiving only minimal information and having low expectations of the health system. Participants expressed a passive acceptance of doing what they were told: *“Well really, I’m just your average Kiwi who thinks that doctors and professionals know best, and if they say “Take this pill”, I take that pill”* (60, NZ European). However, these same patients admitted that they had questions that they had never asked, or information they wanted but never received. *“No, I never ask that question, but I often, in the back of my head think, you know, if I ran out [ …*] *can I get away with it?”* (60, NZ European). There was a feeling of reluctance to challenge and acceptance of the suboptimal service received: *“I suppose it would be handy to have it on a piece of paper, but that just seems to be asking too much of doctors”* (85, NZ European).

## Discussion

This study is the first to explore the medicines information needs of general medicine patients and the barriers and enablers to these needs being met in a hospital setting, using a qualitative methodology. The five themes emerging from the data illustrate both the information needs of participants and the barriers and enablers to receiving medicines information. The theme of autonomy describes the medicines information needs of patients. The themes of fostering relationships, access and communication suggest ways that healthcare providers can optimise the sharing of medicines information; these themes also reflect barriers faced by patients.

The theme of autonomy is consistent with literature and the goals of current health policies, which focus on patient-centred care [22, 23]. This theme highlights that healthcare providers providing individualised medicines information to patients is empowering [23]. The importance of autonomy is reflected in the work of Sheridan et al., who identified the desire for “self-management” when interviewing patients with chronic conditions [24]. This links closely with the NZ Code of Health and Disability Services Consumer’s Rights which includes “autonomy” and being “fully informed” [25]. Additionally, Crossing the Quality Chasm, a report by the Institute of Medicine, highlights patient-centred care as one of six main elements necessary to provide high-quality care [26]. The theme of autonomy reveals motivations behind the specific information that patients’ desire. Understanding why patients want information may motivate healthcare providers to better meet these information needs. Relationships and rapport with healthcare providers facilitated effective sharing of medicines information. This is reflected in previous studies who reported participants wanting to engage with clinicians in a way that allowed conversation relevant to their needs [24].

The findings highlight the importance of access to medicines information through a range of media, though their healthcare provider was the preferred medium as this allowed personalised information. The importance of medicines information being delivered in an individualised manner that considers the social, cultural and physical needs of the patient was highlighted. In particular, the idea that a person’s health is not just an individual issue but a holistic one involving whānau (family) was particularly dominant in some ethnic groups such as in Māori, Pacific and Asian people. This is an important consideration in line with previous literature reporting varying amounts of information and ways of information delivery being desired by patients, but generally highlighting the need to personalise information, with most participants wanting more information than is currently provided [5, 9, 10, 27, 28]. Meeting these needs requires healthcare providers to partner with patients to individualise care and ensure rapport, access to information and providers, including follow up, and effective provider communication skills.

Interestingly, as captured in the theme ‘minimal information needs’, a significant number of participants were accepting of receiving minimum information, which was driven by low expectations of the health system. Whilst some expressed discontent with their standard of care, very few had actively made complaints or changed health providers. This lack of complaint may be influenced by a desire to maintain a relationship with a long-term provider or to appear co-operative or, for some, from the belief that there is nothing that could be done to improve their care [24]. This finding highlights the importance of empowering patients to seek the care and information they desire rather than accepting the status quo, and ensuring system changes to provide patient-centric care as the norm.

These results emphasise the need to support and facilitate healthcare providers to supply medicines information in a way that is individualised to their patients’ needs. Patients want medicines information to be provided by a trusted healthcare provider, at times that suit them, and to be communicated with in a way that maximises their understanding of the information provided.

### Limitations

This study was completed at only one hospital site and participants were drawn from patients admitted to the general medical wards. A combination of age, gender and ethnicity was used to guide the purposive sampling to ensure a diverse representation of participants, however socioeconomic factors were not able to be considered. The influence of social and cultural factors is likely to play an important role in New Zealand on healthcare and medicines information needs; research into how these factors influence patient preferences is warranted. A key strength of this study however is the over-representation of Māori and Pacific peoples within the study; 17% of study participants identified as Māori or Pacific, compared to hospital statistics which report that 8 and 10% of the hospital catchment area identify as Māori and Pacific respectively. However, participants needed to be able to converse in English to participate which may have limited inclusion for some ethnic groups; how generalisable these findings are to all patient groups is therefore unknown. Additionally, social desirability bias may have influenced responses [29]. To minimise this risk, the interviewer was introduced as an independent researcher who was not a member of participants’ healthcare team.

## Conclusion

This study explored the medicines information needs of general medical patients and the barriers and enablers to meeting these needs. The themes of autonomy, fostering relationships, access, communication and minimal information needs were identified. Individual medicines information needs differed, however, the themes identified were common to most and are important factors to consider to help patients understand their treatment and health. Enablers for providing this information were identified as having established relationships with healthcare providers and easy access to both them and other sources of information.

## Supplementary Information

Additional file 1: **Supplement S1**. Discussion guide for patient interviews.

## Data Availability

The datasets generated during and/or analysed during the current study are not publicly available due to potentially identifiable information about participants based on their full transcripts. De-identified data can be made available from the corresponding author upon reasonable request.
