# Supplementary material for: What information do patients want about their medicines? An exploration of the perspectives of general medicine inpatients
Source: BMC Health Serv Res. 2020 Dec 8;20:1131. doi: 10.1186/s12913-020-05911-1 (PMC7722322; doi:10.1186/s12913-020-05911-1)
Supplement: Supplementary file 1 — Supplement S1. Discussion guide for patient interviews. [file 12913_2020_5911_MOESM1_ESM.docx]

**Supplementary appendix S1**

Supplement S1: Discussion guide for patient interviews

| General medicines information   1. Thinking back to a time when you were newly started on a medicine, what information would you have liked about that medicine? |
| --- |
| Timing of medicines information   1. When would you have liked to receive this information about the new medicine that we just talked about? Which aspects would you like at various times? |
| Provider of medicines information   1. Who would you have liked to receive the information about your medicines from? Why? |
| Format of medicines information   1. How or in what format would you have liked the information about your medicine? |
| Use of the internet to obtain medicines information *[reported elsewhere*]*   1. How do you usually access the internet? 2. Have you ever used any websites to get information about your medicine? 3. How often would you use the internet to look up information about medicines? 4. Can you remember some of the websites that you have looked at and used? Did you use search engines (like Google)? 5. When looking up medicines information on the internet, what have you looked for? 6. In general, how do you feel about using web-based (internet) information/resources to obtain information about your medicines? 7. In general, how do you feel about using social media (Facebook, Twitter etc) to obtain information about your medicines? 8. What things might encourage you to use the Internet to find information about medicines? 9. What things stop you from using the internet to find information about medicines? |
| Barriers/enablers to obtaining medicines information   1. In general, what do you think stops you getting the information about your medicines that you need? 2. What helps you getting the information about your medicines that you need? |
| Involvement of family or support people   1. How important is it to you for a family member/s or a support person to be involved in this process? |
| Culture   1. How does your culture relate to your medicines? Would it be useful to have a cultural interface to help you understand your medicines? |
| Conclusion   1. Is there anything you’d like to share about your experiences with receiving/obtaining medicines information that you think would help us to provide a better service to other patients? |

***** Honey MLL, Aspden TJ, Brackley KE, Haua R, Sheed L, Ashmore-Price H, et al. Patients' internet use in New Zealand for information about medicines: Implications for policy and practice. *Health Policy and Technology*. 2018;7(2):119-24.
